# Supplementary material for: Biotin proximity tagging favours unfolded proteins and enables the study of intrinsically disordered regions
Source: Commun Biol. 2020 Jan 22;3:38. doi: 10.1038/s42003-020-0758-y (PMC6976632; doi:10.1038/s42003-020-0758-y)
Supplement: Supplementary file 1 — Supplementary Information [file 42003_2020_758_MOESM1_ESM.docx]

**
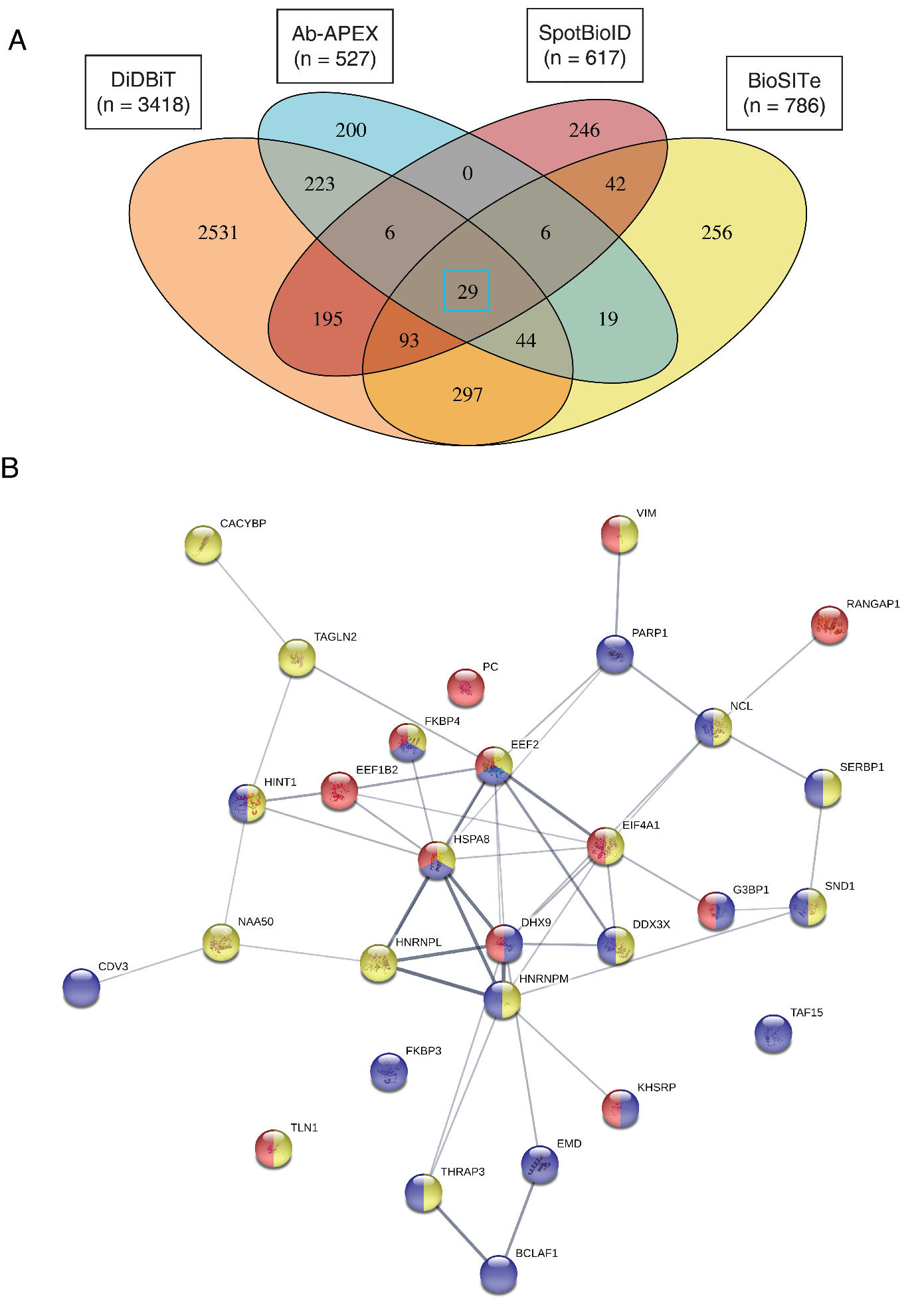
**

**
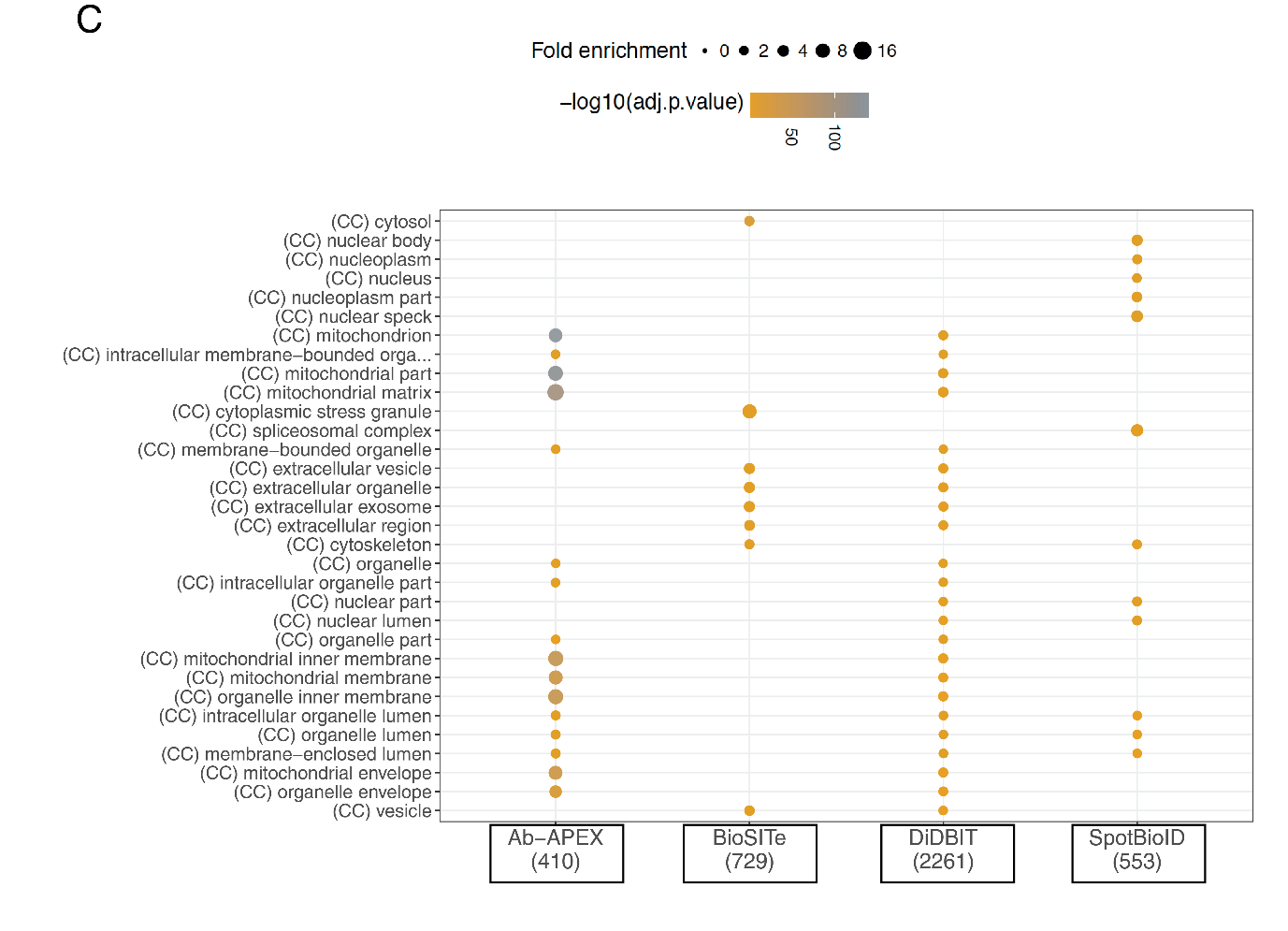
**

**Supplementary Figure 1.** Functional themes in overlapping proteins.

1. A Venn diagram showing the overlap of proteins across the four studies used in this analysis. There is a very small number of proteins (29) common to all 4 studies (blue box). DiDBIT has the most number of exclusive proteins as it targets the entire cell. Ab-APEX targets the mitochondrial matrix and inner mitochondrial membrane. SpotBioID targets the nucleus and BioSITE targets the cytoplasm. (B) A connectivity plot showing any published and validated interactions between the 29 common proteins identified in Supplementary Figure 1A. This image was generated using the online program STRING (<https://string-db.org/cgi/input.pl?sessionId=QqkSTNv1EVki&input_page_show_search=on)>.An enrichment analysis was run on the 29 proteins using Gene Ontology (GO) categories and the colours represent some of the most significant terms from this analysis. Blue indicates that these proteins are known to localise to the nucleus, red indicates localisation to the cytosol and yellow indicates localisation to the extracellular region. Multiple colours in a single circle indicate that the given protein has been found in multiple locations in different studies. (C) GO Cellular Component enrichment analysis for the 4 studies. The proteins in each study were mapped to GO:CC categories and compared to a background of the published HEK293 proteome which was also mapped to GO:CC categories. The size of the dot represents the fold enrichment over the background, i.e. the fraction of proteins in the input list that are annotated by a given GO term divided by fraction of proteins in the background list that could be mapped to the same GO term. The colour of the dot represents the significance of the enrichment with grey being most and orange being least significant. Note that all terms displayed in this figure are significant and above the adjusted p-value cut-off of 0.05.

**
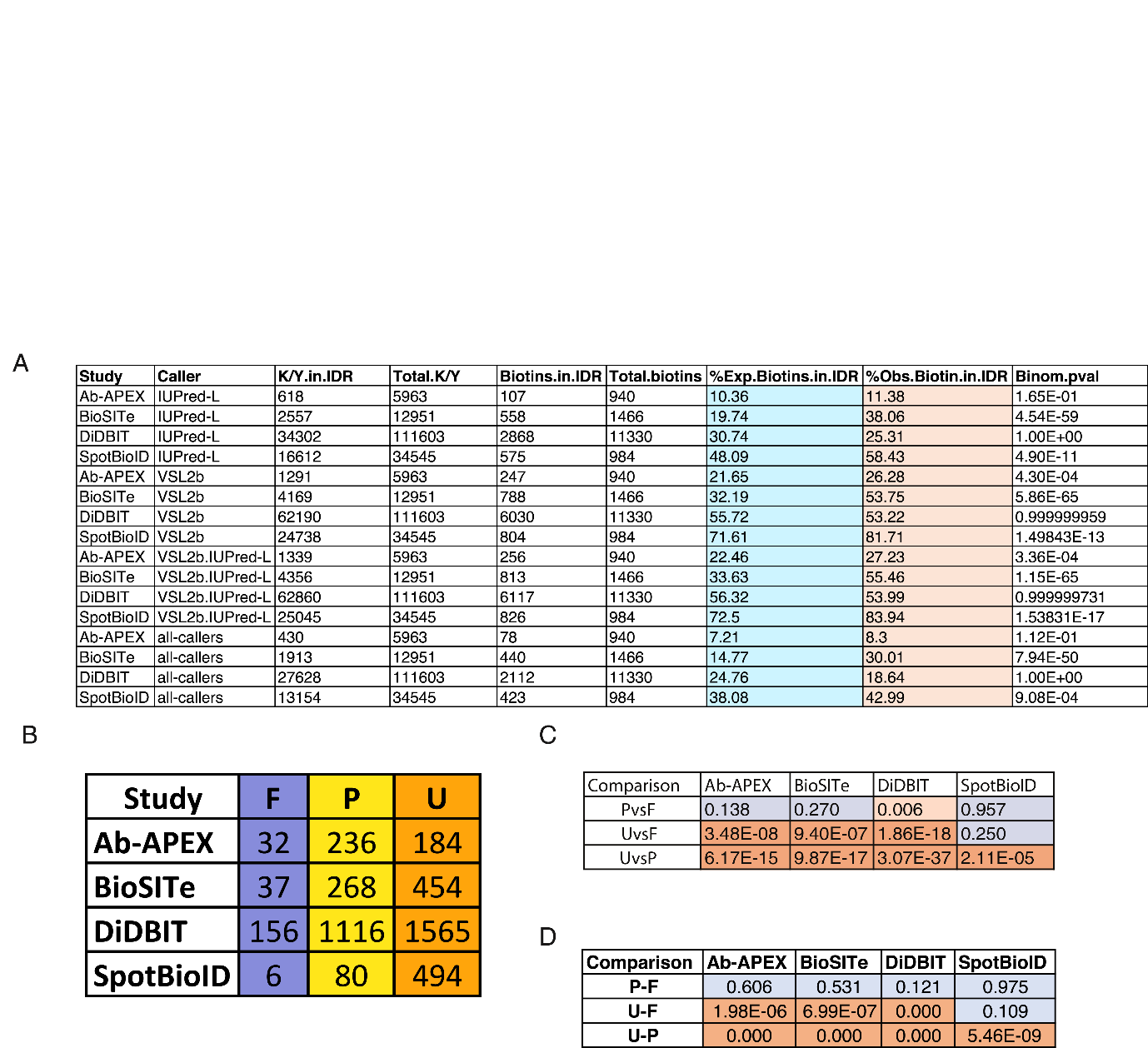
**

**Supplementary Figure 2.** Statistics for Biotin and IDR comparisons.

(A) Table of counts and percentages used to look at the Expected (Blue) and Observed (Orange) rate of biotin occurrence within IDRs across all studies and all callers. The last column “Binom.pval” denotes the p-value using a bionomial test where the “probability of success” is the (Target residues in IDRs/Total Target residues), a “success” is a biotin within an IDR (Biotins.in.IDR) and “number of trials” is the total number of biotins (Total.biotins) observed in that study. All tests are significant at the p = 0.05 level. We have not imposed a ‘correction factor’ for any putative differences in accessibility between IDRs and folded proteins as the *in vivo* accessibility of IDRs is unknown in most cases. K = Lysine; Y = Tyrosine (B) A table displaying the frequencies of proteins in each of the IDR categories in each of the 4 studies using the IDR predictor VSL2b. To test whether or not there were significant differences in the number of biotins found in regions of IDR across the 3 IDR categories (C) a pair-wise t-test with multiple testing correction between the three groups – F (Folded), P (Partially Folded) and U (Unfolded). The table shows the p-value of these pairwise t-tests across the four studies. Light blue denotes comparisons that are not significant. Light orange denotes significant (p<0.05) and dark orange denotes comparisons that are highly significant (p << 0.05) (D) an analysis of variance (ANOVA) was performed followed by a Tukey Honestly Significant Differences (THSD) test to correct for family wise error. The table shows the p-value of the THSD test across the four studies. Light blue denotes comparisons that are not significant. Light orange denotes significant (p<0.05) and dark orange denotes comparisons that are highly significant (p << 0.05)


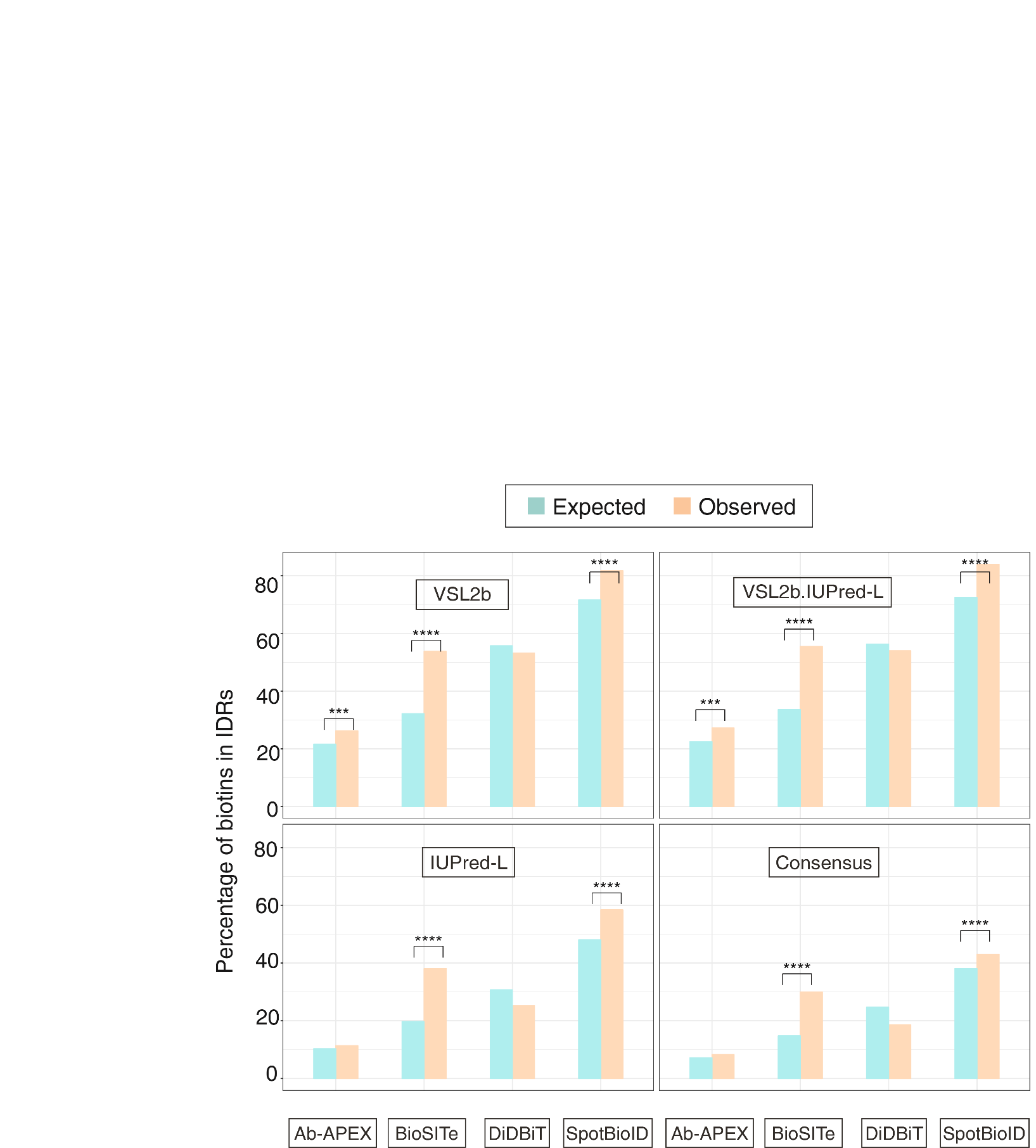


**Supplementary Figure 3.** Comparison of IDP predictions.

Barplots showing the Expected (pale blue) and Observed (pale orange) distribution of biotins within regions of IDR across the 4 studies using 4 predictors (bottom). ‘VSL2b.IUPred-L’ means that a region is denoted as being an IDR if called so by one or both algorithms (union). ‘Consensus’ means a region is denoted as being an IDR if called so by all algorithms listed in the D2P2 software at the time of analysis.

**
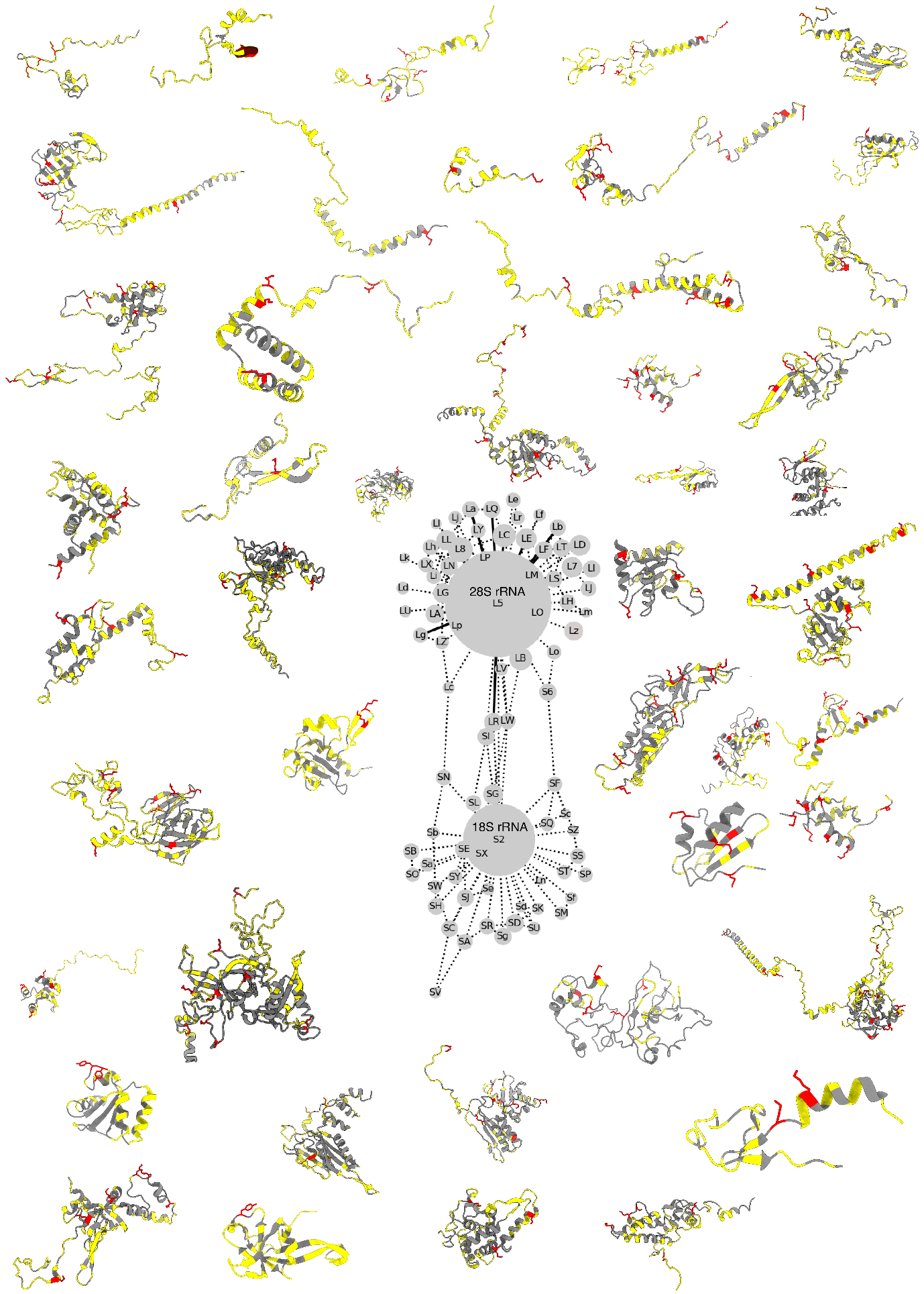
**

**Supplementary Figure 4**. Sites of *in vivo* biotinylations mapped on in silico disassembled 80S ribosome (PDB: 6EK0).

An “exploded” ribosome plot showing the individual proteins that make up the eukaryotic 80S ribosome in ribbon representation. Biotinylation marks from four biotinylation datasets are highlighted in red. We can see that nearly all ribosomal proteins are biotinylated. Extensive biomolecular interfaces (between proteins and ribosomal RNA hubs as well as protein-protein interfaces) are highlighted in yellow. The central graph summarises the large number of interactions between individual ribosomal proteins and the 28S and 18S ribosomal RNA molecules.


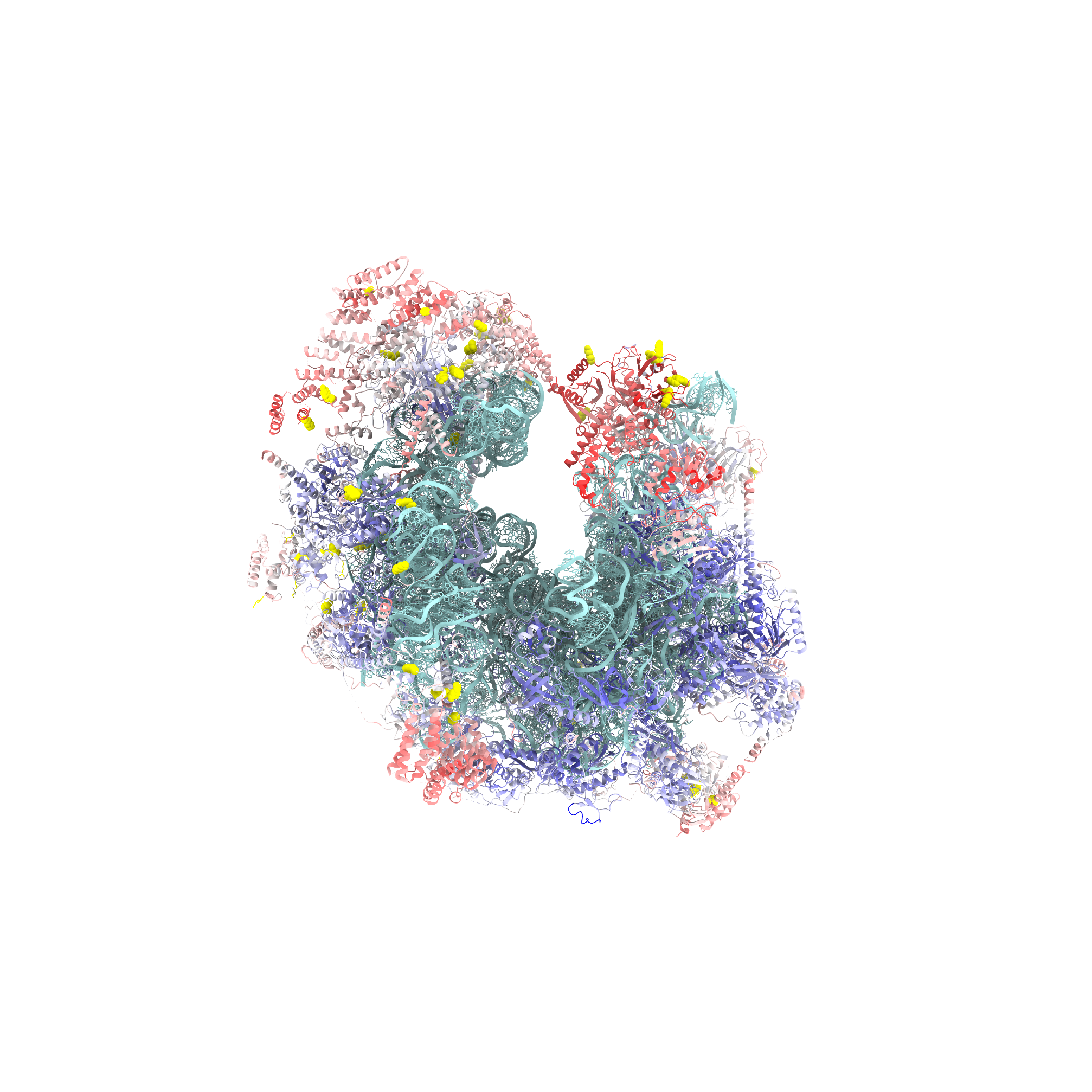


**Supplementary Figure 5.** Sites of *in vivo* biotinylations from DiDBiT and Ab-APEX studies mapped on the human mitoribosome (PDB: 3j9m).

Mitoribosome cryo-EM structure in ribbon representation with sites of biotinylation highlighted in yellow and full-atom spheres and rRNA in pale turquois, proteins colored by B-factor (blue= minimal; red=maximal; B-factor is also referred to as ‘displacement factor’ and is a measure of local mobility).


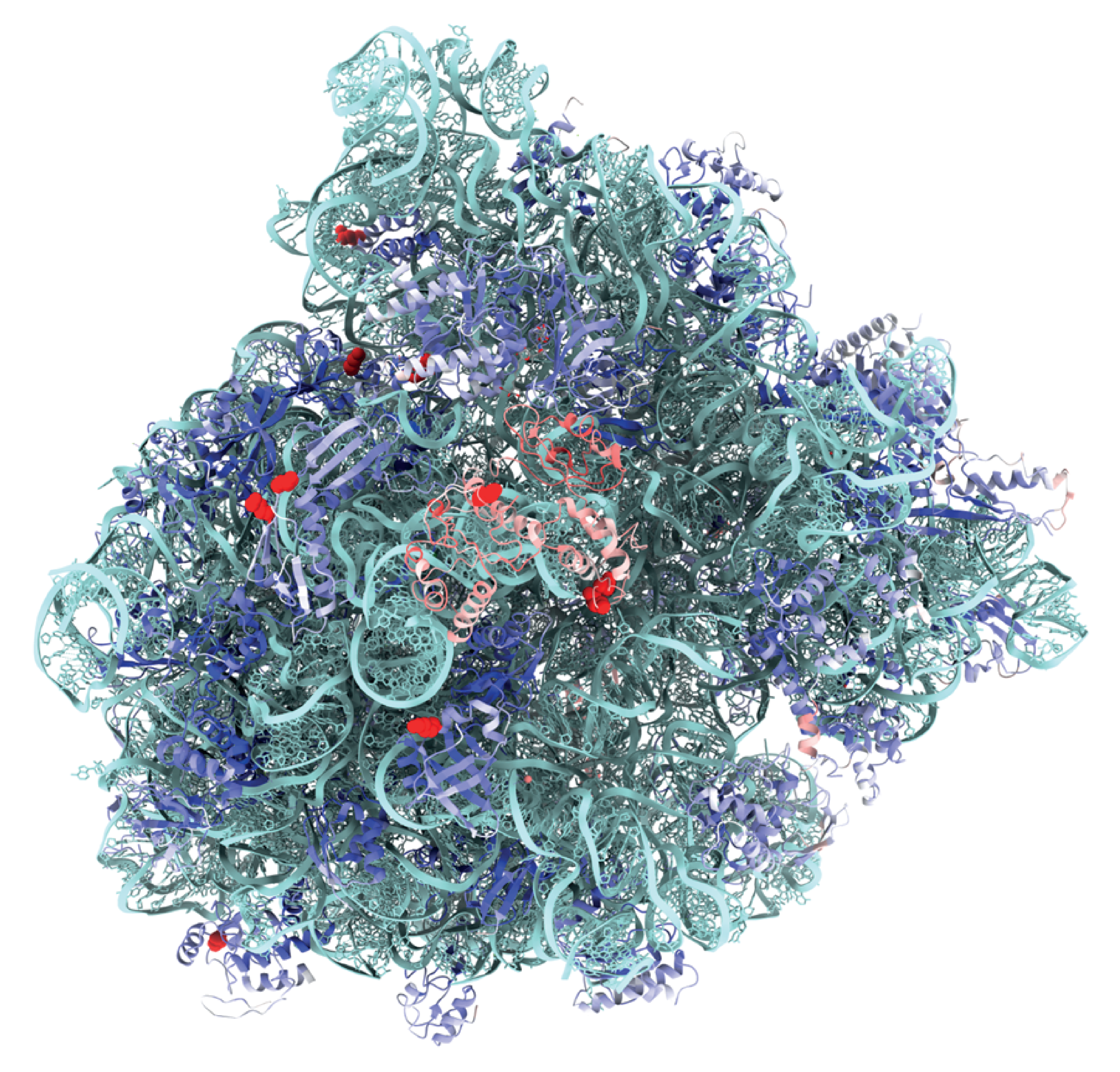


**Supplementary Figure 6**. Early sites of *in vitro* biotinylations mapped on the 70S ribosome (PDB: 5afi) after ten seconds.

70S structure in ribbon representation with sites of biotinylation highlighted in red and full-atom spheres and rRNA in pale turquois, proteins coloured by B-factor (blue= minimal; red=maximal; B-factor is also referred to as ‘displacement factor’ and is a measure of local mobility).


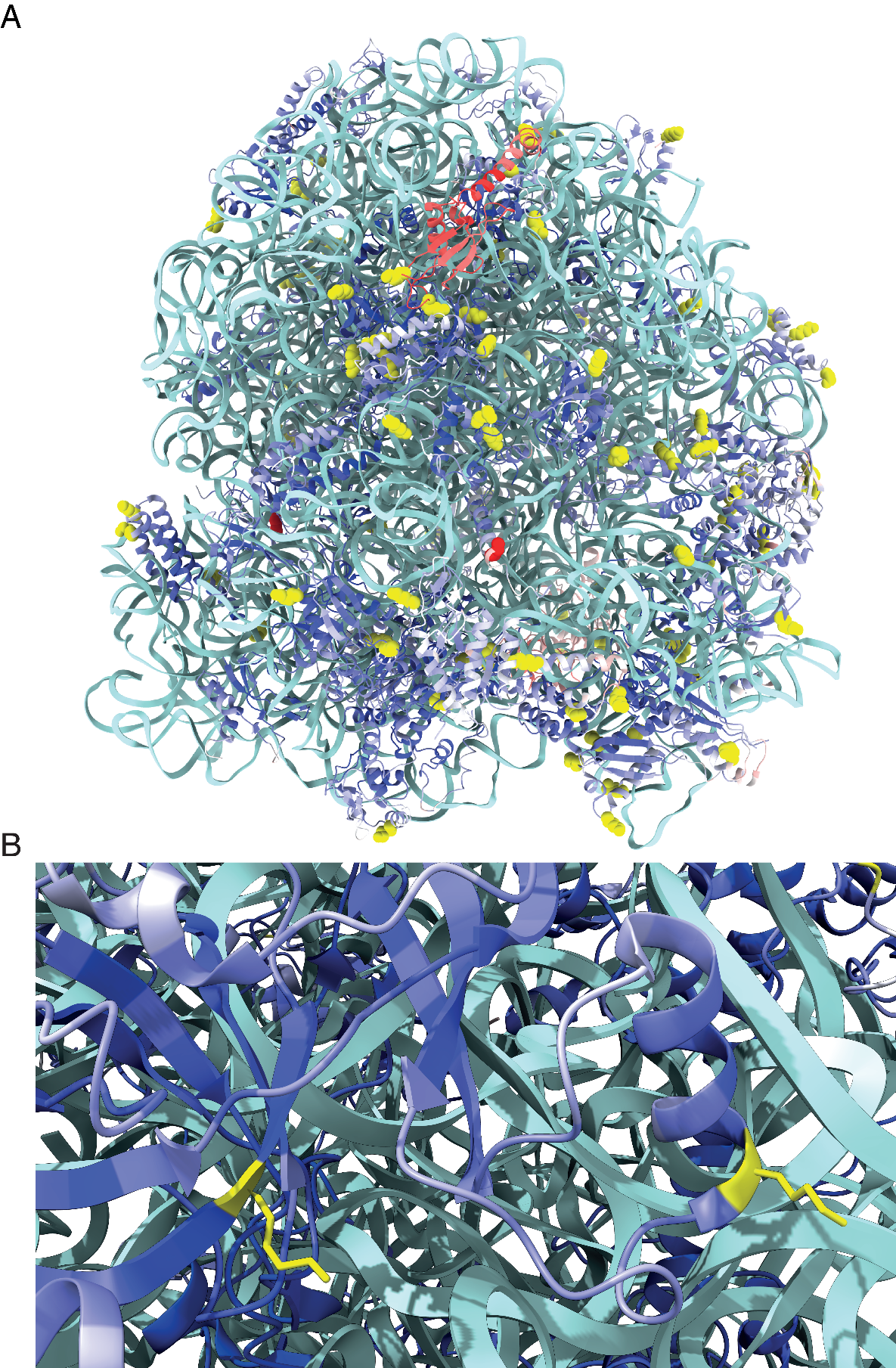


**Supplementary Figure 7**. Late sites of *in vitro* biotinylations mapped on the 70S ribosome (PDB: 5afi) after five minutes.

(A) 70S structure in ribbon representation with sites of biotinylation highlighted in yellow and rRNA in pale turquois, proteins coloured by B-factor (blue= minimal; red=maximal). (B) L3 slowly biotinylated examples in structured regions.


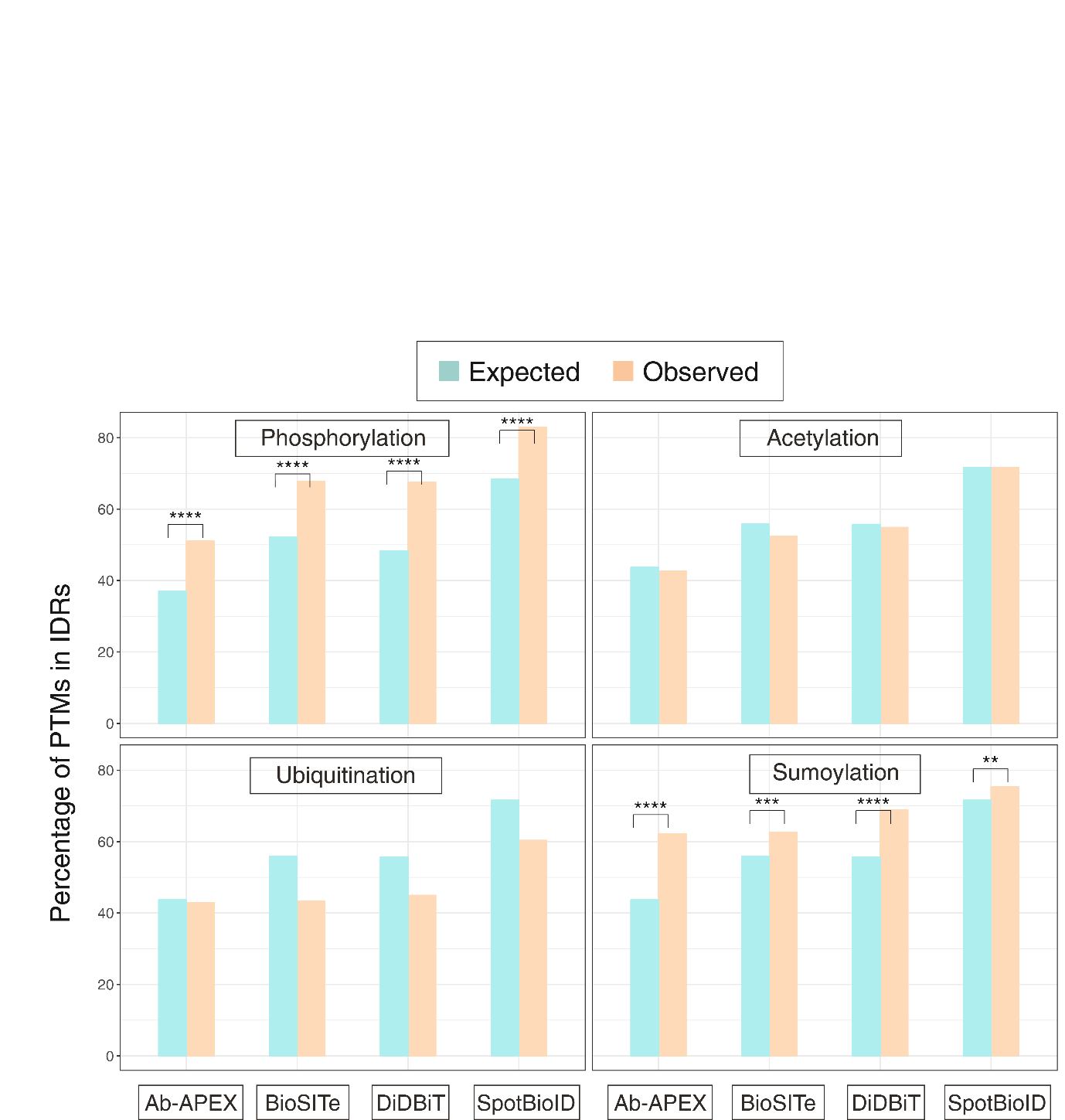


**Supplementary Figure 8**. Correlation between IDRs and post-translational marks.

Barplots showing the Expected (pale blue) and Observed (pale orange) distribution of PTMs within regions of IDR across the 4 studies – Phosphorylation, Acetylation, Ubiquitination and Sumoylation. There were significantly more Phosphorylation and Sumoylation marks being identified in IDRs than expected based on the rates of occurrence of S, T, Y and K in the peptide sequences used in the analyses. Statistics that accompany this figure are included in Supplementary Data 1.
