# Supplementary material for: Biotin proximity tagging favours unfolded proteins and enables the study of intrinsically disordered regions
Source: Commun Biol. 2020 Jan 22;3:38. doi: 10.1038/s42003-020-0758-y (PMC6976632; doi:10.1038/s42003-020-0758-y)
Supplement: Supplementary file 2 — Description of Additional Supplementary Files [file 42003_2020_758_MOESM2_ESM.docx]

**LEGEND FOR SUPPLEMENTARY MOVIE**

**Supplementary Movie 1. *In vivo*** biotinylation sites within the human 80S ribosome. Specific residues that have been biotinylated in proximity studies are highlighted in orange in the context of a high-resolution cryo-EM structural model of the human 80S ribosome (PDB ID 4v6x). Ribosomal RNA is shown virtually transparent.

**LEGENDS FOR SUPPLEMENTARY DATA**

**Supplementary Data 1.** Source data for Figures 2,3,4 and Supplementary Figures 1,2,3,4,5,8.

**Supplementary Data 2.** Source data for discussion of subcellular consensus across four studies, HPA, LOPIT and Uniprot assignments.

**Supplementary Data 3.** Source data for Supplementary Figure 6 and Supplementary Figure 7.
